# Multiple-target tracking in human and machine vision

**DOI:** 10.1371/journal.pcbi.1007698

**Published:** 2020-04-09

**Authors:** Shiva Kamkar, Fatemeh Ghezloo, Hamid Abrishami Moghaddam, Ali Borji, Reza Lashgari

**Affiliations:** 1 Machine Vision and Medical Image Processing Laboratory, Faculty of Electrical and Computer Engineering, K. N. Toosi University of Technology, Tehran, Iran; 2 Brain Engineering Research Center, Institute for Research in Fundamental Sciences (IPM), Tehran, Iran; 3 HCL America, Manhattan, New York City, United States of America; MIT, UNITED STATES

## Abstract

Humans are able to track multiple objects at any given time in their daily activities—for example, we can drive a car while monitoring obstacles, pedestrians, and other vehicles. Several past studies have examined how humans track targets simultaneously and what underlying behavioral and neural mechanisms they use. At the same time, computer-vision researchers have proposed different algorithms to track multiple targets automatically. These algorithms are useful for video surveillance, team-sport analysis, video analysis, video summarization, and human–computer interaction. Although there are several efficient biologically inspired algorithms in artificial intelligence, the human multiple-target tracking (MTT) ability is rarely imitated in computer-vision algorithms. In this paper, we review MTT studies in neuroscience and biologically inspired MTT methods in computer vision and discuss the ways in which they can be seen as complementary.

## Author summary

Multiple-target tracking (MTT) is a challenging task vital for both a human’s daily life and for many artificial intelligent systems, such as those used for urban traffic control. Neuroscientists are interested in discovering the underlying neural mechanisms that successfully exploit cognitive resources, e.g., spatial attention or memory, during MTT. Computer-vision specialists aim to develop powerful MTT algorithms based on advanced models or data-driven computational methods. In this paper, we review MTT studies from both communities and discuss how findings from cognitive studies can inspire developers to construct higher performing MTT algorithms. Moreover, some directions have been proposed through which MTT algorithms could raise new questions in the cognitive science domain, and answering them can shed light on neural processes underlying MTT.

## Introduction

We are an intelligent, creative, and efficient species able to perform a large amount of processing in a fraction of a second. For many years, artificial intelligence (AI) researchers have tried to replicate these capabilities in machines. However, AI uses different approaches than the brain uses to fulfill this goal. One of these approaches focuses on general biological systems for inspiration. Some successful methods include: ant-colony optimization (ACO), inspired by how ants find their path in a colony; the bees algorithm (BA), inspired by the honey bee food-foraging method; the genetic algorithm (GA), inspired by natural selection in evolution; particle swarm optimization (PSO), inspired by the movements of birds in a flock or fish in a school; and the artificial immune system (AIS) algorithm, inspired by the vertebrate immune system. These methods are widely used to solve a variety of problems in the field of AI ([1] reviews bioinspired methods).

To develop algorithms based on the brain, we first need to understand how it works, i.e., how the brain performs particular tasks and the exact mechanisms underlying a particular behavior. Second, we need to imitate brain functions in order to create algorithms that make computers capable of performing a similar task. This approach has led to some publicly known and powerful tools, such as artificial neural networks (ANNs) and its variations (e.g., deep neural networks [DNNs], inspired by the hierarchical layers of neurons in the brain; convolutional neural network [CNNs], inspired by the concept of receptive fields; and spiking neural networks [SNNs], inspired by how neurons contribute to each other via spike trains). Some areas of research, such as reinforcement learning (RL), incremental learning, attention, and saliency detection, have also emerged to imitate brain functions, although the exact approach they use may not completely align with the processes used by the brain. In addition to understanding how the brain solves a particular task, some studies have focused on modeling the entire brain [2,3].

Visual information is one of the most important sensory inputs used to perceive and interact with the environment. To fulfill all of its duties, our visual system must accomplish some basic tasks, which can be combined to define more complex functionalities. These tasks correspond to basic challenges in computer vision, such as object detection and recognition, object tracking, and activity recognition. This paper focuses on object tracking as a key part of different applications, such as video surveillance systems, video understanding, or human–computer interaction.

Although object tracking in computer-vision literature usually refers to tracking a single object in a video [4,5], MTT is something done by humans in the real world. Two-year-old babies can track more than one target [6]. As they grow, human ability to track more objects simultaneously increases [7]. Different examples in daily life include monitoring children on a playground or in a swimming pool, tracking multiple vehicles and pedestrians while driving a car, or watching a basketball game. MTT is a well-known paradigm in neuroscience; after [8], a pioneer study on the human ability to track multiple visual targets, many researchers investigated this phenomenon to identify the brain areas involved [9–11] and understand the factors influencing it [12]. Most of these methods use psychophysical experiments and behavioral data analysis, but in recent years more advanced techniques (such as functional MRI [fMRI]) have been utilized as well to uncover the neurobiological bases of MTT.

Two approaches are used in computer vision for MTT. The first defines multiple instances of a single-object tracker (SOT) and assigns each instance to one target independently. Here, the same strategy is used to track multiple objects, and cognitive neuroscience studies of single-object tracking can be helpful. The second approach develops an MTT algorithm that can track more than one object simultaneously. This approach is advantageous since it can benefit from the shared information in the whole system, which is useful for tracking individuals and handling challenges. In general, the MTT algorithms in computer vision are still far from the exact mechanisms employed by the brain, even the brain-inspired algorithms. These brain-inspired algorithms mainly consider the key cognitive effects that undoubtedly play a role in MTT, such as attention and memory. However, there is much more to learn from the cognitive neuroscience of MTT.

**Fig 1:**
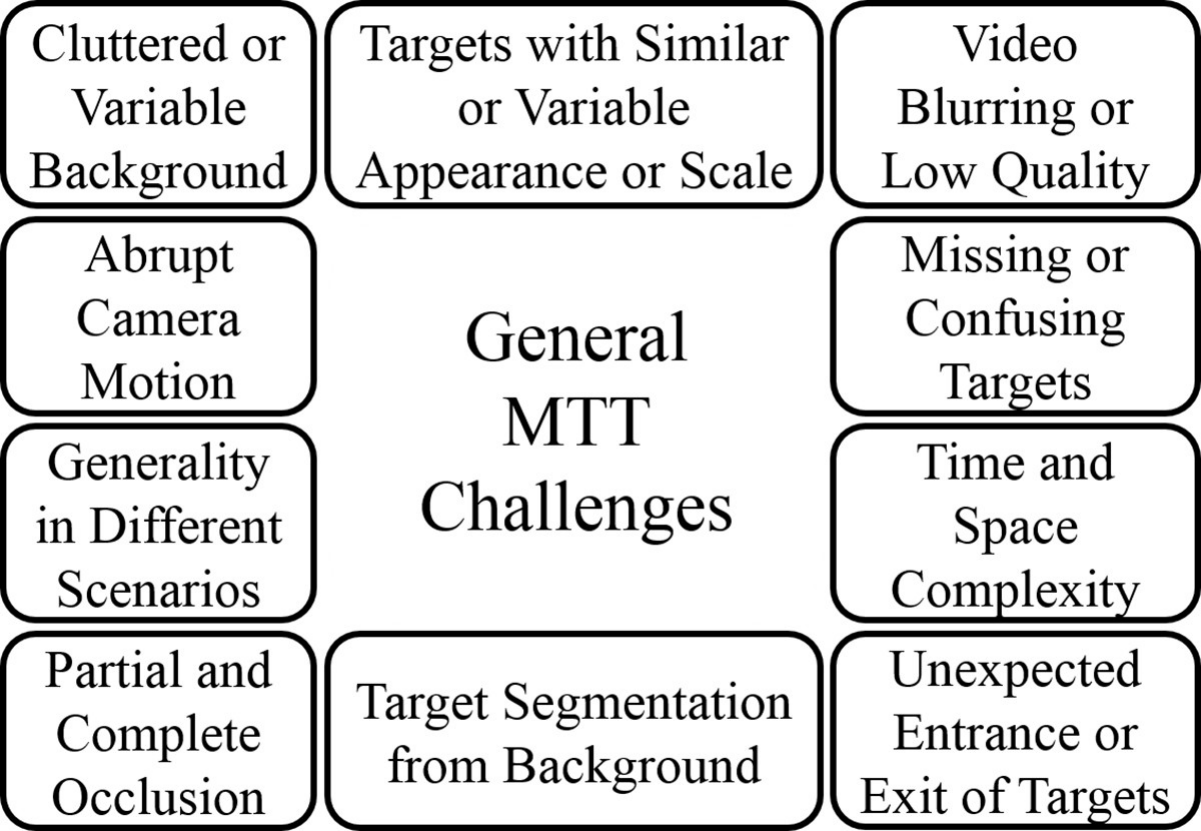
MTT general challenges. MTT, multiple-target tracking.

It is worth noting that the brain chooses an efficient strategy to track multiple targets considering its physical and cognitive limitations; these limitations are mostly related to the physiological properties and functions of neurons, which are tuned to specific features of visual scenes, visual spatial mapping, and varied emotional and intrinsic brain states [13–17]. Computers do not have similar computational limitations. Therefore, what would be the advantages of using a brain-inspired approach to achieve high performance MTT algorithms? First, considering human-brain limitations, algorithms’ performance is still lower than human performance, especially in challenging scenarios [18]. For MTT, humans still perform more accurately than current MTT algorithms as long as the number of targets is less than four items (because of human cognitive limitations [19]). However, human performance depends on factors such as object spacing too [20]. Humans are very efficient at tracking multiple objects from different categories and very robust to image transformation, whereas algorithms have to be trained each time over a new data set. In this respect, algorithms lack generalizability. Algorithmic performance is evaluated with respect to ground truth. Ground truth for an MTT application contains exact positions of targets in each video frame. In fact, in most cases, humans generate ground truth directly or via some annotation tools that streamline the process. However, state-of-the-art MTT methods remain unable to detect all targets in all frames, even offline methods that have sufficient time and information to process the video. This is likely due to various challenges that a typical MTT algorithm must overcome (Fig 1).

Second, developing algorithms with low computational cost is integral to the computer-vision domain. Such algorithms can be more easily customized for embedded systems and real-time applications. Thus, investigating how the brain allocates its limited resources efficiently and performs in different situations can help the field of computer vision develop higher performing MTT algorithms that require less computational and memory resources.

Despite substantial progress in the areas of neuroscience and cognitive science, the exact strategies exploited by the brain to solve problems such as MTT are still not completely understood. However, studies have shed light on some parts of these strategies and raise the following questions: Are all types of MTT studies in the field of cognitive neuroscience useful to improve computer-vision algorithms? We believe the answer is no. Current brain-inspired MTT algorithms use cognitive factors proven to be effective in experimental studies. For example, an attention or memory module can be added to an algorithm to imitate the role of visual attention in feature extraction or memory in retaining extracted information. Some experimental studies in the field of multiple-object tracking (MOT; see the “MTT Studies in Neuroscience” section) investigate how humans allocate their limited attentional resources to multiple moving objects in the scene. However, overt attention cannot be assigned to more than one point at any time. What is the mechanism behind selecting that point? How do humans suppress unrelated information to avoid being sidetracked so they can track targets successfully? Is using one hemifield as beneficial as using both for object tracking? On the other hand, computers have sufficient computational resources to get high-resolution information from all points of the screen. They can also consider both related and unrelated information on the screen at the same time without any distractions. Therefore, these types of studies are likely not as beneficial for increasing tracking algorithm accuracy although they might help to find a solution for the effective use of computational resources in the proposed algorithm. Some cognitive experimental studies have investigated different challenges that humans face, such as partial or complete occlusion, targets with variable appearance, and crowding. These challenges were the topic of a significant amount of research in the computer-vision domain [21], but they remain unsolved. Therefore, investigating human behavior could prove to be the most efficient and effective way to design algorithms to solve MTT problems.

Despite some potential issues, computer vision can help define specific MTT challenges. Unlike experimental studies (performed only on simple artificial scenarios), algorithms are used in real-world situations. Therefore, they confront various challenges with which humans are already familiar. Studying human behavior while tracking multiple objects in real scenarios can help to uncover normal strategies used by the brain. The results of such research will be more beneficial than research on simple artificial scenarios used in cognitive neuroscience of MTT.

In sum, MTT, as an important paradigm in neuroscience, involves a variety of applications in computer vision. There are consistencies and inconsistencies between studies in these two areas. Some of these are due to intrinsic differences between the human brain and computational algorithms. But others are likely due to a research gap between these two potentially interrelated fields. Here, we mention some of the consistencies. Human behavioral studies have shown that memory and attention are cognitive processes that are undoubtedly involved in MTT [22]. Several current proposed algorithms benefit from modules with similar function, although their implementation details may not be entirely consistent with how the brain works. For example, Mahadevan and Vasconcelos suggested that salient items are tracked easier by humans [23]. Some automatic methods also use discriminative features and saliency detection to track objects. Furthermore, some neuroimaging studies have reported activation of areas responsible for object recognition in the brain during the tracking of multiple targets [24]. Similarly, several MTT algorithms use pretrained object-detection modules to find targets in each frame and track them.

Still more findings from MTT cognitive studies are worth noting for the design of algorithms. For example, it is reported that any surface features or semantic information that leads to visual separation between targets and distractors can improve the performance of subjects during tracking [25–28]. However, no MTT algorithm in computer vision has utilized this finding as inspiration for its methods. It could be incorporated by processing both semantic and surface information of targets in an object-recognition module in MTT algorithms, which may improve tracking performance. Object-detection methods have recently benefited from including semantic features [29,30]. Some MTT algorithms have also utilized such methods to detect objects (“MTT Methods Based on ANNs” section); however, they discarded semantic relations between targets. This additional information increases discriminability of targets from other objects and may improve performance of MTT algorithms. As another example, human vision is binocular, and it has been reported that humans perform better when depth information is also available in MTT stimuli [31]. However, there are still open questions about how and to what extent human binocular or even monocular depth vision influences the handling of challenges such as clutter, scale variance, and data association or occlusion. Most MTT algorithms assume monocular vision. Some SOTs improve tracking performance utilizing a binocular camera [32]. Improving MTT performance using a similar idea is anticipated. Indeed, this issue has rarely been considered by both communities. However, the efficiency of methods based on binocular vision compared to monocular vision in other applications (such as object detection) has been proven [33]. On the other hand, there are challenges that target-tracking algorithms confront; these include the variability in target appearance and background, partial or complete occlusion, the birth and death of a target, and data association in real scenarios. It is still not clear exactly how the brain performs in such situations, but cognitive studies can focus on these topics to understand the brain in more detail. Even drawing inspiration from this incomplete survey may improve both accuracy and runtime of automatic MTT algorithms.

In the “MTT Studies in Neuroscience” section, we review the most recent ideas about MTT from the neuroscientific community. We discuss brain-inspired MTT methods in computer-vision area in the “Brain-Inspired MTT Algorithms” section. Finally, in the “General Discussion” section, we describe commonalities and differences in these two domains and explore how these two veins of research can be mined to better understand the mechanisms behind MTT and to develop stronger computer-vision algorithms.

## MTT studies in neuroscience

**Fig 2:**
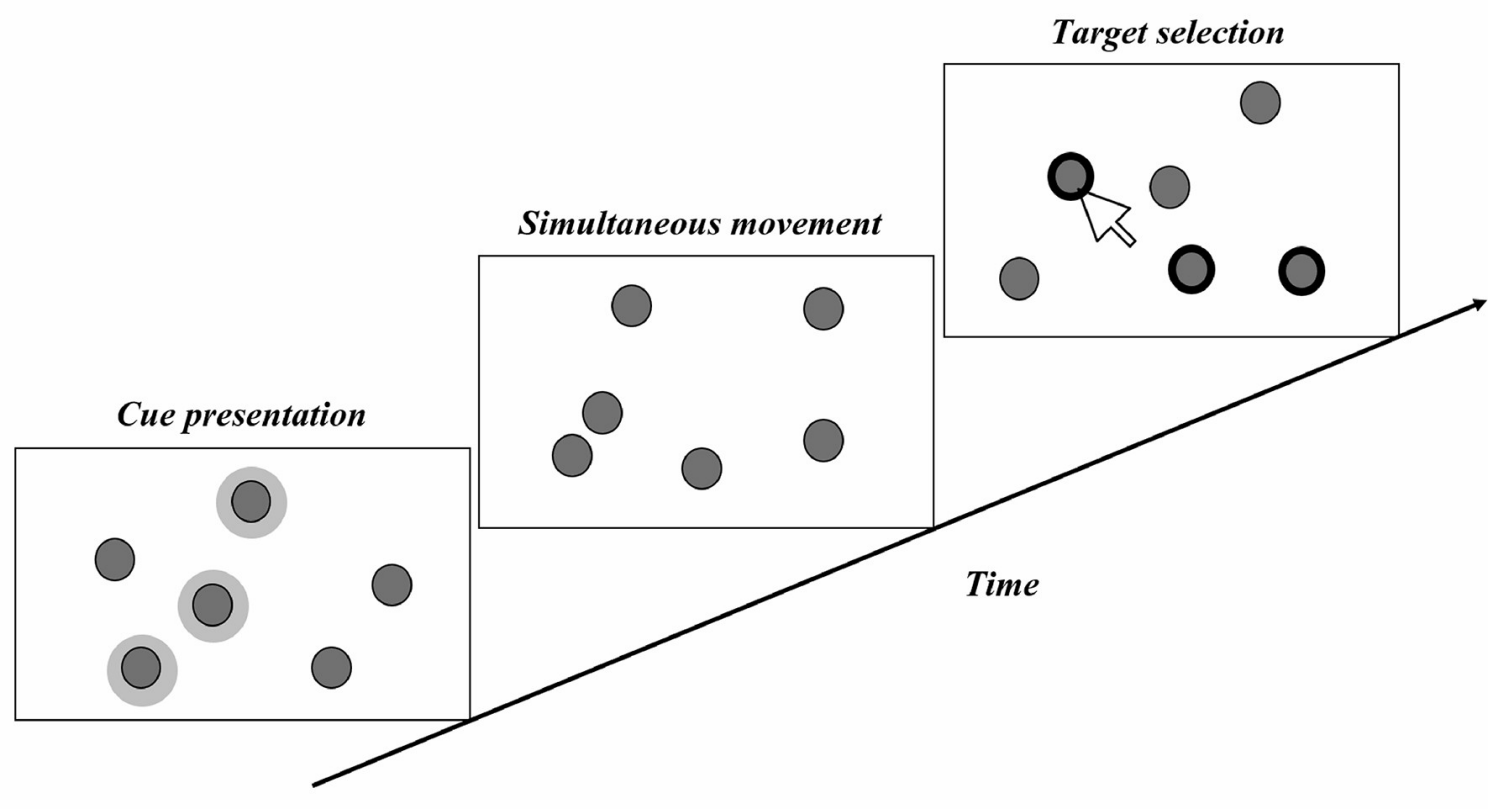
The Basic MTT paradigm.

Researchers in the field of MTT investigate how human subjects perform during psychophysical experiments. Fig 2 shows the classic MTT psychophysical task, which begins by introducing targets to be tracked among distractors. Then, both targets and distractors move simultaneously for a random period of time, and subjects are finally asked to identify the target(s). The final step can be done in two ways: (1) A probe is shown, and subjects are asked whether it was one of the targets, or (2) participants are instructed to select all of the targets among all stimuli. There are also two methods for evaluating the performance of each subject. The first is calculating the ratio of the correct trials to the total trials done by subjects and reporting the value as their performance. A correct trial is a trial in which the subject marked all targets correctly. The second method involves computing the ratio of correctly marked targets to the total number of targets in a trial and then calculating the mean of these values over all trials of a subject. Finally, the average performance of all subjects is reported as human performance in the task.

Many factors can vary in this task, such as the number of targets and distractors; the speed, type, and duration of movements; and the choice of targets and distractors. Decisions on these parameters are defined by the research objectives for individual studies. It is common to divide MTT tasks into two types of experiments: MIT (multiple-identity tracking) and MOT. For MIT, the task involves tracking objects with different identities among dissimilar distractors. MOT involves tracking similar targets when there is no difference in the appearance of targets and distractors.

### MIT versus MOT

Tracking multiple targets with different identities (done in MIT) differs from tracking indistinguishable targets (done in MOT): MIT requires both recognizing targets’ identity and remembering their location. It is postulated that, generally, there are two distinct mechanisms involved in MTT [12,34]. In [12], subjects were required to track multiple indistinguishable objects in one experiment and multiple objects with different identities in another experiment (instances of MOT and MIT, respectively), and their performances were compared between the two task conditions. It is reported that during MOT, people remember only location information and forget target features since they are not informative [12]. However, in MIT, both the feature and location of the targets are important, and subjects had to bind both pieces of information together to successfully complete the task. Thus, it was proposed that MOT is a parallel process, and MIT is a serial one. Analyzing the eye movements of the subjects helps better investigate this; if MOT is a parallel mechanism, the subjects should rarely look at the targets, while if MIT is a serial mechanism, subjects should focus on targets one after another. Recently, a review of eye behavior studies for both MOT and MIT is provided in [35].

**Fig 3:**
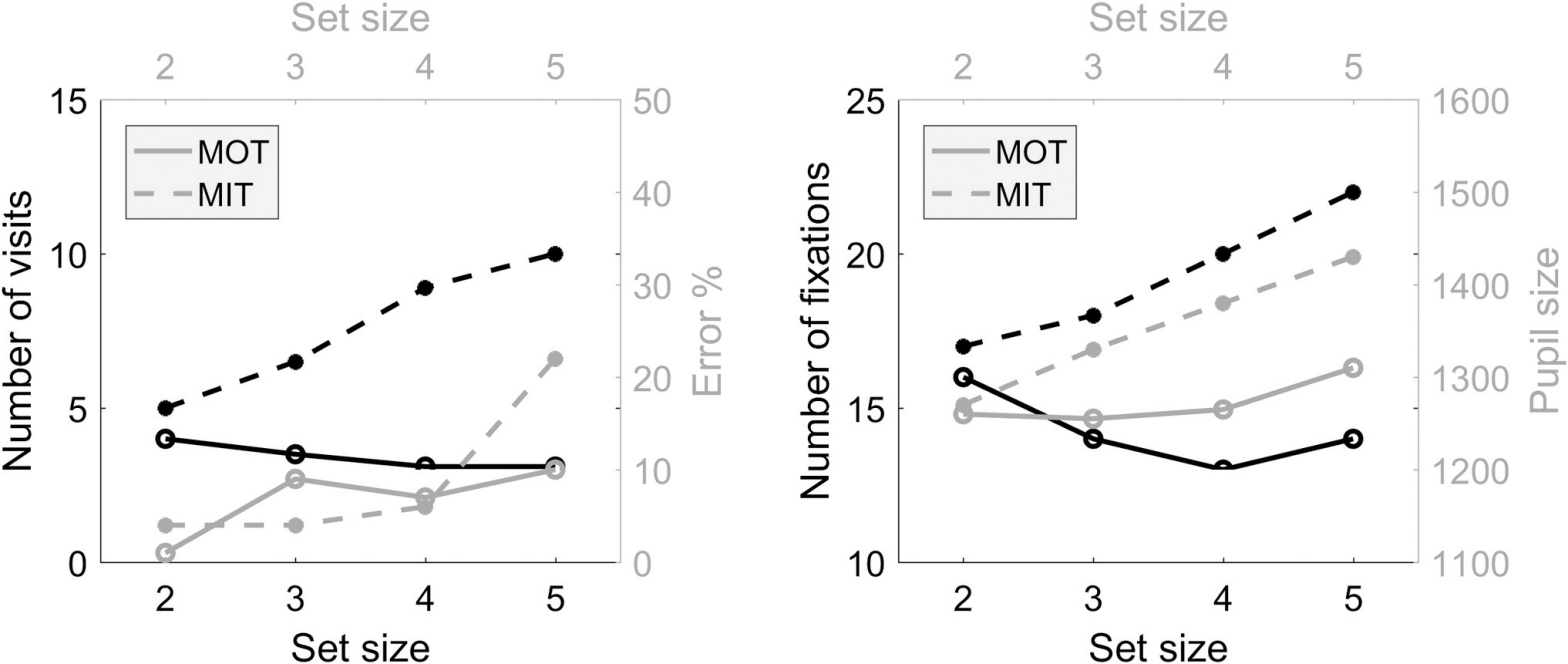
Comparing subjects’ performance in MOT and MIT tasks using the results from Experiment 3 of [12]. MIT, multiple-identity tracking; MOT, multiple-object tracking.

In [12], the number of targets visited as well as the number of fixations, (i.e., fixation frequency) are recorded. The pupil size and blink rate were also quantified as a measure of attentional load. The authors manipulated the target-set size (number of targets) and speed of the objects during the task and studied the effect of these manipulations on the two recorded parameters. They postulated that MOT occurs in parallel, since their results showed that several parameters, such as the number of target visits and fixations, were not affected by target-set size. Subjects showed overt attention near to one target and tracked the others covertly. Pupil size, a measure of attentional load, increased with the number of targets. Fig 3 shows some results from Experiment 3 of this study. In this experiment, authors used black- and white-line drawing pictures to design one MOT task and one MIT task. The same group of subjects performed both tasks. The fixation map covered approximately the center of the screen in MOT. Slowing down the target's speed of motion led to serial tracking; an increase in target-set size in the slow-speed condition resulted in an increase in the number of target visits and fixations. For MIT, the number of target visits and fixations should increase with set size. In fact, subjects fixated more on the targets but with shorter duration as target speed increased, which supports serial tracking in MIT. Additionally, eye-tracking data showed that all targets are tracked overtly by the subjects, and tracking performance for recently visited targets is higher than targets that were visited earlier [36]. The fixation heatmap covered nearly the entire screen area since the objects move randomly.

The authors also found that pupil size was larger in MOT than MIT. It is believed that there are two systems responsible for MTT [12]: One ambient system is responsible for tracking the positions of objects, and another system is focal and responsible for recognition of individual items. These two systems must then be bound together to enable MTT. In MOT, only the former is used, while in MIT both the former and latter are used. Therefore, the mechanisms underlying MOT and MIT are neither completely independent nor the same. When the subjects are instructed to track identities, location tracking is impaired [37]. That is due to inherent limitations of cognitive resources such as attention and memory. Studies have further postulated that MIT and MOT mechanisms share common resources, and this allocation process is flexible [37–41].

Some studies provide evidence contrary to the aforementioned findings. According to [42], the number of fixations stays the same with the increase in the target-set size for both MIT and MOT. It is postulated that subjects use the same strategy to perform both tasks. They designed a different experiment to investigate the matter. Subjects were instructed to track moving objects with the speed of 6 deg per sec for 8 seconds. For MIT, the objects were cartoon-animal images, and, after the cue, they were covered by gray circles during the tracking phase; the objects finally stopped, and a probe was shown. Afterwards, an animal appeared at the center of the screen, and the subject was asked whether the probe was that animal or not. It is worth mentioning that MIT is commonly referred to as those tasks for which the identity of targets and distractors are visible during movements. Therefore, the aforementioned results can be regarded in line with other findings suggesting that tracking indistinguishable objects needs just location information and is parallel, while tracking distinguishable targets is serial [12].

A recent study suggested that MIT performance manifests a more serial pattern when high-resolution information is required, and a relatively parallel pattern when low-resolution information suffices for tracking [22]. Authors recorded eye movements and behavioral responses of subjects during tracking multiple distinct faces from the same sex, disks with different colors, and distinct line drawings in separate tasks. The eye-movement results showed a more serial pattern when tracking faces and drawings and a parallel pattern when tracking colors. That is, when tracking faces and drawings, participants visited each target serially, while when tracking color discs, more eye visits to blank areas between targets were observed. When high-resolution information is needed for identifying a target, foveal vision is directed to it; on the other hand, when low-resolution information is sufficient for discriminating the targets, the eyes tend to land on blank areas to sample information from multiple targets covertly [22]. MTT algorithms usually use a serial procedure to track multiple targets. Leveraging the advantages of parallel programming, it is possible to recruit different cores of a processor and assign each core to track one target. This decreases the algorithm’s runtime and makes it more suitable to apply for real-time applications. However, a limited number of algorithms benefited from this strategy [43].

To pinpoint the neural circuits responsible for binding target identity and location during MIT, fMRI and eye-tracking data were analyzed simultaneously in [24]. Areas responsible for working memory, attention, and object recognition were active in these tasks, and the authors postulated that binding is performed in lateral frontal and ventral occipitotemporal areas. Identity switching during a tracking task to investigate which part of the brain is responsible for binding object identity and location is the goal of another study [44]. Three different scenarios were considered: 1) switching the targets’ identity, 2) switching the distractors’ identity, and 3) no identity switching. The fMRI data were acquired while the subjects were tracking multiple circles tagged with different letters. Identity switching was applied by changing the letter on the related object. Results show that targets’ tag switching was associated with activity in the dorsal attention network (frontal eye fields [FEF] and intraparietal sulcus [IPS]).

### Main topics in MTT

Although classic MTT tasks can be considered either MOT or MIT, many recent studies customize classical experiments to answer their particular questions. Therefore, it is difficult to label them either a pure-MIT or a pure-MOT task. However, these customized versions are more similar to MIT since they rarely use the same objects. In fact, pseudo-MIT scenarios are ubiquitous in daily life in which there are usually some visual features that help the observer distinguish among objects, specifically tracked and nontracked ones (e.g., tracking different color clothing while also tracking multiple children on a playground or attending to the model of vehicles being tracked while driving). Most computer-vision problems also involve issues with real-world applications, so MIT or pseudo-MIT studies are the main focus of this paper. MOT scenarios in which all objects are completely similar rarely occur in practice. However, studying these tasks helps to better understand the brain mechanisms underlying MIT. In this paper, we also tap into some MOT studies if their findings are useful for empowering algorithms. For a review of MOT studies, please refer to [45,46].

#### The effect of semantic information

Semantic difference between target and distractor categories facilitates MIT [25]. In [47], authors attribute this facilitation to the categorical distinction of targets and distractors, supported by four processes. First, any type of visual distinction between targets and distractors facilitates tracking. Second, semantic differences in targets and distractors require the attentional system to choose a different strategy for distributing attention to objects and thus facilitate tracking. Third, categorical information might be saved in visual working memory and thus improve the error recovery process. Fourth, there is a mechanism that groups information according to its category, leading to easier tracking of targets among distractors that belong to different categories. The authors conducted six experiments targeting this hypothesis and found that visual distinctiveness is not the only reason that differential category membership of targets and distractors induces facilitation in tracking performance: Intercategorical information is also important. Their results rejected the “change in attentional distribution strategy” hypothesis since targets always attracted more attention than distractors. Eye-tracking data have shown that fixations are directed toward targets; the frequency of fixations landing on the targets increases with enhancing the attentional need for processing the targets [36]. This is why change detection is also easier for targets than distractors in a tracking task [48]. Finally, they argue in favor of the fourth process underlying this effect, i.e., the categorical distinction reduces the interference of the distractor in target tracking, so there is a semantic category-based system that facilitates tracking in similar situations. This suggests that adding common features between targets as shared information may increase MTT algorithms’ performance and improve their accuracy in discriminating targets from other parts of the scene. Effectiveness of a similar idea was proved in the object-recognition domain [49].

To understand which brain area is responsible for semantic category-based grouping, brain activity using fMRI is studied [9]. Subjects in the experiments tracked multiple targets in three scenarios: 1) All targets and distractors were from the same category, 2) targets and distractors were from two distinct categories, and 3) half of the targets and half of the distractors were selected from one category and the others from a different one. Results suggested that the fusiform and pars triangularis of the inferior frontal gyrus are involved in semantic categorization.

Familiarity of targets also facilitates tracking. One can regard this familiarity effect as a kind of semantic distinction. Tracking multiple familiar identities versus tracking unfamiliar ones is compared in [26]. It is postulated that the ease of tracking multiple familiar identities originates from less cognitive load needed to remember the identity of targets. Relating this to the two systems responsible for MIT, location and identification, familiar targets reduce cognitive load since identification of these targets is less cognitively demanding. Familiarity of objects depends on the frequency of their use in trials of a block of experiments. To study familiarity, the identity of targets and distractors are the same in all trials within a block. In the unfamiliarity condition, the identity of targets and distractors always change, and there are no two trials with the same targets and distractors in the block. In [50], authors performed an experiment to investigate whether among familiar objects (animals and man-made ones) there is any bias toward one category or the other. They asked subjects to track multiple objects among animals and artifacts. Although they observed a small increase in identity accuracy for animal targets, they suggested that animal targets do not improve location tracking and that there is no absolute advantage in their tracking over man-made objects. The neural processes involved in tracking familiar targets and unfamiliar ones are examined in [26]. While tracking unfamiliar objects, activity in regions within the attention network, and areas responsible for identification increase. However, activity in areas underlying memory performance increases while tracking familiar objects. Accordingly, it is suggested that MIT is a two-stage process: finding the location of targets in the first stage and identifying them in the second stage. When subjects localize the targets, they extract some key features to identify them and understand what is where. As the subject becomes progressively more familiar to targets, key features can be determined more efficiently while ignoring irrelevant features. This process facilitates finding the loci of targets.

Emotional content of the target items can also affect tracking performance. Li and colleagues performed a multiple–identical-face tracking task to examine the effect of facial emotion on tracking performance [27]. Subjects could track angry faces as targets more successfully than other faces in general. This effect persisted even when another angry face existed among the distractors. This means that processing of emotional expression happens quickly and affects MTT performance. They did not report such effects for a happy expression.

Familiarity and emotion can make both targets and distractors semantically distinct. This type of distinctiveness facilitates perceptual grouping and increases MTT-tracking performance. In fact, discriminating targets from nontargets is easier when utilizing both semantic and appearance information compared to appearance only. Computer-vision algorithms consider appearance features to discriminate targets and distractors, but semantic information has rarely been used for this purpose. Using semantic information improved an algorithm’s performance in an object-detection application [29,30]. Applying a similar approach to MTT algorithms can be beneficial and potentially improve their performance. In addition to semantic categorization, the visual features of objects can also affect MTT accuracy. For example, assigning a single color to all targets and another color to all distractors makes tracking easier [28]. In the following section, we explain how appearance features affect MTT.

#### The effect of surface features

Surface features such as color and shape are the most easily perceivable features that help an observer distinguish between objects. A study investigated whether surface-feature information contributes to MOT or if tracking multiple objects is based on spatiotemporal information alone [51]. The experiment consisted of several spheres moving on a floor plane. The floor plane was presented against a black background from a viewpoint angle of 20°. Target spheres were introduced by flashing red at the beginning, after which they started moving. In the meantime, distinct colors were assigned to the spheres for a period of time. An abrupt scene rotation around the axis occurred after motion onset. At the end of the trial, participants had to select the target objects. Their results indicated that brief presentation of object colors around a scene rotation influences tracking performance, and distinct color matching across the scene rotation improved tracking. Swapping the distinct colors after rotation impaired tracking performance. The authors introduced a flexible-weighting tracking account, revealing that spatiotemporal information and surface features are both utilized by the location-tracking mechanism. The two sources of information are weighted according to their availability and reliability. Surface-feature effects on tracking are particularly likely when distinct surface-feature information is available and spatiotemporal information is unreliable [51].

To investigate the extent to which tracking can be improved by surface-feature distinctiveness in object identities, in another experiment subjects are asked to track a subset of objects either unique in color or unique in color and shape [52]. Results showed that tracking is feature based, and there is a limitation in feature binding. Tracking performance improved when objects were distinct in shape or color; however, when the targets were distinct from one another, tracking was not enhanced if distractors shared the target’s colors or digit identities. This finding contrasts with previous studies, where participants were found to have poor memory for surface properties in a MOT task [53]. Participants use a strategy for tracking to set up a competition between the two types of perceptual grouping: grouping on the basis of attentional set (tracking targets versus nontargets) and grouping on the basis of surface features (red objects versus green objects and so on). This was also reported in [54]: When targets and distractors have distinct features, only surface features of targets are maintained in visual working memory; when targets have the same color as distractors, they are more difficult and consume more attentional resources to track.

In [28], authors investigated how much uniqueness of object identities affects tracking accuracy. They asked participants to track four targets among eight objects that were: (1) identical in color (homogeneous condition), (2) of different colors (all unique; red, green, blue, yellow, orange, azure, brown, and pink), or (3) represented by two or four total colors (heterogeneous condition). They found that accuracy was affected by heterogeneity of tracked objects, and color uniqueness of targets and distractors enhanced performance. The similarity between targets is not important; rather, it is the distinction between targets and distractors that is important. This was further shown in [55], by investigating separately the impact of chromatic and categorical distinctiveness of colors on tracking. Circles from two green and blue categories in different hue ranges were used as objects in the proposed MTT task. Results revealed that the varying distinctiveness in color between the targets and distractors significantly influenced tracking performance. Also, tracking accuracy was affected by the chromatic difference between targets and distractors, which may be attributed to the effect of perceptual grouping [55]. Further, in a separate study, fMRI data showed that the putamen and temporoparietal junction (TPJ) may be involved in this color-based grouping [56]. In [57], authors investigated to what extent target-distractor differences in terms of color, contrast polarity, orientation, size, shape, depth, and combination of shape, color, and size influences tracking accuracy. They compared accuracy in these conditions to accuracy in situations in which two targets and distractors share one feature and the others share another feature. Tracking performance was always better in the former case. Results showed that subjects use a range of features to highlight target-distractor differences that facilitate tracking.

Surface features such as color and shape are encoded in early visual-processing areas of the brain [58]. According to the preceding findings, these features undoubtedly influence the processing of targets and distractors during MTT and facilitate tracking. It seems possible that there is a flexible feature-selection method in our brain that selects a minimum number of discriminative features to separate targets and background in general. However, MTT algorithms lack generality and are biased to find the best feature set for only one specific application. We next study how low-level feature and depth influence tracking motion.

#### The effect of motion features and depth

Motion information is also important in MTT [59]. To investigate motion information’s effect on visual attention through MOT, a task that added motion to the texture of each object and to the background is designed [60]. The texture inside targets either stayed stationary or moved relative to the direction of the target's motion. This motion could be in the direction of a target’s motion, opposite to the motion of the target, or orthogonal to it. Tracking accuracy was better in the same direction condition than the orthogonal condition and better in the orthogonal condition compared to the opposite condition. These results show that texture motion influences tracking accuracy, but texture speed does not affect it. Thus, when there is a motion conflict between the target’s motion direction and the texture motion, tracking is impaired. This result was also replicated in a 3D scenario. Intrinsic motion of an object is a feature that is not used by MTT algorithms, although this feature could help to discriminate nonrigid and deformable targets from other parts of a scene.

In addition to intrinsic motion, an object’s movement speed and direction are usually set randomly to force the object to have independent motion trajectories in MTT tasks. In some studies, velocity is considered constant to ignore the effect of acceleration [61]. In addition to tracking in a 2D environment, a limited number of experiments were performed on 3D MTT tasks that found an advantage to tracking in 3D compared to 2D; for example, it has been reported that stereopsis effect has a positive impact on MOT [62]. It can even facilitate some related skills similar to playing football. Tracking multiple objects in a 3D environment improves passing decision-making accuracy in soccer players [63]. Using depth information in computer-vision algorithms is beneficial, too [32,33], even though MTT algorithms rarely use binocular vision and depth information.

Simple low-level surface or motion features involve less attentional resources compared to high-level information, which can have a negative impact on MTT performance. In the following section, we focus on how identity processing of objects interferes with location processing.

#### The interference of identification

The common resources used for processing location and identity information cause them to interfere with each other [37,64]. In one study, subjects were asked to track four objects from eight identical circles and then select targets and report their identity at the end of each trial. Results showed that tracking identical targets is easier than recalling their identities. It is possible that subjects have an internal name for targets paired with external labels; however, this also assumes that internal names are available for use outside the tracking task. It is also possible that the internal name is available only for the purpose of tracking and is not retained outside that process. This would operate similarly to a local variable in a computer subroutine, which is not available to the program that calls the subroutine [64]. In [65], authors studied to what extent we can recognize identity while tracking multiple objects. They asked subjects to track grayscale human faces with neutral expressions from a Chinese face database. Study results indicated that processing of target identity is a mandatory process that can occur even when it is task irrelevant. Results also showed that performance of tracking different faces was impaired compared to tracking of identical faces, which can be explained by a dynamic identity-location binding for faces and objects by some mandatory processes. The study also demonstrated that tracking and identity processing share the same attentional resources.

When high-level processing such as face identification is involved in MTT, the identification process requires more attentional and memory resources, resulting in lower MTT accuracy and location information processing. In fact, people tend to use optimized features that not only can discriminate targets better but are also encoded and retrieved faster and more easily. This is why in the real world, where a lot of information exists for processing, we are still successful in tracking various objects simultaneously. Some MTT algorithms benefit from an object-detection module to determine all targets in all frames of video. Some use discriminant features to separate targets from background and track them in next frames. However, the brain utilizes a combination of these approaches. We have thus far considered how visual information affects identification and location processing in MTT; the following section explores the influence of other information, such as spatial configuration.

#### Spatial configuration and the hemifield effect

To study the role of overall spatial configuration of targets in MTT performance, an MIT experiment is designed in [10], and subjects are asked to track objects with either distinct colors or distinct irregular shapes. They found that the spatial configuration of targets impacts their identification. Preserving the form of a nonrigid polygon (with each target as a vertex) while tracking, the targets can be identified more accurately. This suggests that subjects tend to mentally build a polygon, considering each target as a vertex. They focus on its center of mass overtly and track the vertices covertly. This means that the responsibility for tracking targets is assigned to both hemispheres, which offers a benefit of use in MTT algorithms, such as tracking cars in a highway. Specifically, this strategy could help to detect the location of objects that are partially or completely occluded. Hemifield effect is also studied during MIT [66]. Comparing tracking performance when all targets are moving in one hemifield versus when they are distributed in both hemifields showed that the cognitive resources for tracking are not hemifield specific. Tracking accuracy is higher when targets are distributed across two hemifields, and this bilateral advantage was stronger during MOT than MIT. Tracking is partially independent in two visual hemifields, and the degree of independence is greater in MOT than MIT.

In [67], the authors investigated the effects that speed and proximity of objects to each other have on tracking performance. They used the Planets and Moons Tracking (PMT) paradigm, which consists of a series of dots rotating around local centers and also around a fixation marker, similar to a solar system. Some factors of the experiment (proximity, speed, eccentricity, and number of distractors) can be manipulated independently. The results of this study clearly indicate that speed, proximity, and the number of distractors in the display each influence tracking performance while the other factors are held constant. Thus, models of object tracking need to include mechanisms that are sensitive to these factors. These results suggest that there are at least two mechanisms at play during tracking: One mechanism is sensitive to the number of distractors in the display, while a second is sensitive to target-distractor proximity as well as the speed of the objects in the display [67]. Tracking performance declines with increasing speed and decreasing distance between objects. Overall tracking accuracy is always higher in 3D compared to 2D [68]. According to [69], more attention is allocated when targets are in a crowded situation and have a higher chance of being lost. More precise tracking is needed in such situations, and an increase in accuracy in these cases supports the idea of dynamic allocation of attentional resources.

#### Occlusion and crowding

Studies have shown that humans are able to track targets successfully even if targets move behind an occluder or are out of view (due to disappearance) for a short time [70–72]. Although occlusion causes performance to decrease slightly compared to the no-occlusion condition [72], attentional resources are still devoted to occluded or invisible objects [73]. In [74], authors compared brain activities recorded using fMRI while tracking occluded and fully visible objects covertly to unearth neural substrates underlying occlusion handling. They reported that cognitive strategies and mental states behind these two cases differ; they found more activation in four regions of the brain in the occlusion condition compared to no occlusion: inferior parietal lobule, superior temporal sulcus, presupplementary motor area, and precentral sulcus.

Subjects track more successfully when objects simultaneously disappear rather than when they disappear asynchronously for several hundreds of milliseconds and reappear again [70]. Some studies have shown that humans use available and reliable surface features in addition to spatiotemporal information to handle occlusion [51]. To investigate how humans use spatiotemporal features to track objects through occlusion, authors in [75] defined a wall in their MTT task and manipulated location, motion direction, and the side of objects’ reappearance when they go behind the wall and disappear. They reported that MTT performance is better when the target reappearance position is near to its disappearance location regardless of motion direction and whether objects reappear on the same side they disappeared on. In [61] also, authors found that accuracy is higher when objects reappear near their disappearance locus than when they appear on their trajectories. However, a debate persists on whether humans use trajectory information to handle occlusion. Evidence supports both ideas [46]. Authors in [76] argued that extrapolation and motion information are used more frequently when tracking two targets and less frequently while tracking four targets. In the computer-vision domain, most MTT methods use a motion-estimation module, which significantly increased the accuracy of algorithms.

Crowding is another important factor in an MTT task. When the number of objects increases in the scene, the distance between objects decreases. This factor is the primary human limitation in MTT [20]. Performance falls by decreasing spatial separation between targets [77], and in such situations, when the possibility of confusing targets increases, it is suggested that human subjects benefit from rescue saccades (the saccades toward the targets that are in a critical situation) to avoid wrong target association [78]. Studies have shown that subjects utilize working memory and allocate more attentional resources to crowded parts of a scene [69,79]. In addition, they tend to locate their gaze close to the targets that are in a crowd. This way, the crowd is projected to the fovea and can be handled easier because of the fovea’s higher spatial resolution [80]. In general, this spatial limit in MTT is distinct from the attention-capacity limit [81].

## Discussion

Thus far, we have reviewed the main topics on the cognitive neuroscience of MTT. These studies can open up avenues for empowering computer-vision algorithms. For example, research shows that semantic distinction between targets and distractors facilitates tracking. Using object-detection algorithms is common in tracking algorithms, but they usually focus on targets and detect them in the frames of video. However, utilizing more powerful object-recognition tools to detect all existing objects (moving or stationary) helps localize the targets better and avoid missing targets or introducing distractors instead of a targets by mistake. In addition, considering the semantic relation of targets provides more information and helps avoid any confusion between targets and other items in the scene. Object-detection methods have recently benefited from including semantic features [29,30]. Familiarity is also reported as useful.

To use its advantages in computer vision, a memory module in the algorithm can save different appearances of different targets. We can cross-check the similarity of the current candidate with the items saved in memory. This can help recover correct indices, especially when target identification is difficult. A similar strategy was exploited successfully in [82] to model the background of video. This is consistent with activity increases in memory-related parts of the brain while tracking familiar objects. Although using surface features, such as shape, is common in algorithms for both object detection and discrimination from background, an object’s intrinsic motion information is rarely used for discrimination. This feature is particularly useful for tracking nonrigid objects or avoiding wrong data association. The spatial configuration of targets also helps humans track multiple objects. This was not previously considered in algorithms. There are some real-world scenarios in which targets have specific spatial relations (e.g., tracking cars on highways). Although this configuration changes over time, it still improves performance, especially when the target is missed due to partial or complete occlusion or when reporting the exact location of the vehicle is difficult. In addition, cognitive studies have shown the positive effect of tracking in 3D. This shows the benefit of adding another feature, depth, which can help in target discrimination. Using this feature in algorithms can be beneficial, too. Using more features usually increases the opportunity to discriminate and track targets successfully.

The next section reviews existing brain-inspired computer-vision algorithms in MTT. These algorithms benefit from approaches used by the brain to improve performance. We categorize these studies according to their approach and discuss how they can be beneficial for the cognitive neuroscience of MTT.

### Brain-inspired MTT algorithms

Tracking multiple targets automatically remains an important problem in computer vision [83,84]. It is crucial for many applications, such as sport analysis [85–87], biology [88], surveillance [89,90], and human–computer interaction [91]. There are various strategies to track objects in MTT algorithms. One strategy uses an object-detection algorithm to find all candidate objects in each frame and then determine the new loci of objects according to the result of a motion-prediction module. Instead of object detection, another strategy uses discriminative features to separate objects from their surrounding background. Some MTT algorithms are offline, and others are online methods. The latter are suitable to apply to real-time applications.

Regardless of the strategy behind an algorithm, specific metrics are used to evaluate them [92]. One metric, for example, measures the amount of displacement between the targets’ loci determined by the algorithm and real locations of targets. Another metric evaluates whether the location determined by the algorithm is bounded to the correct borders of the objects or whether it contains some parts from another target or unrelated parts of the environment as well. There are several reviews on MTT methods in computer vision [5,83]. Among various methods in this area, a few MTT algorithms try to imitate how humans perform MTT. They used only general findings of cognitive neuroscience on MTT, such as the fact that attention and memory are two important cognitive components that are undoubtedly involved in MTT. Some algorithms are based on ANNs, and we considered them as bioinspired methods, too.

In this section we review MTT algorithms that mimic brain functions in their design. Table 1 compares some of these algorithms according to results reported in their related papers on MOTChallenge data sets (https://motchallenge.net/) with MTT related metrics, such as MOT accuracy (MOTA), MOT precision (MOTP), false positive (FP), and false negative (FN). Data sets used to evaluate computer-vision algorithms are videos recorded from natural environments, which can motivate cognitive studies of MTT to use real stimuli instead of artificial ones. MOTA considers all types of errors that the tracker makes. MOTP shows the ability of an algorithm to determine the exact position of objects [92]. A higher value for MOTA and lower one for MOTP show that the algorithm has high accuracy (i.e., a low number of errors) and good localization. FP shows how many times the algorithm reported a wrong item as an object. FN counts the times that the algorithm was unable to report an existing object [93]. Therefore, low values for both FP and FN are desirable. Some research mentioned in this section involves brain-inspired SOTs. In the computer-vision domain, it is possible to extend such algorithms to track multiple objects, which is their rationale for inclusion in this section.

**Table 1:** Comparison of several discussed brain-inspired algorithms.

Data set	Method	MOTA	MOTP	FP	FN
**MOT15**	[99] (tested on six sequences)	43	74	682	2,780
	[110]	19	71	11,578	36,706
	[98]	34.3	70.5	5,154	34,848
	[113]	37.1	71	7,034	30,440
**MOT16**	[98]	46	74.9	6,895	9,117
	[113]	47.3	74	6,375	88,543

Abbreviations: FN, false negative; FP, false positive; MOTA, multiple-object–tracking accuracy; MOTP, multiple-object–tracking precision; MOT15, multiple-object tracking 15 data set; MOT16, multiple-object tracking 16 data set

We classify existing brain-inspired studies into three categories: attention based, memory based, and methods based on neural networks. These categories are not distinct, and some algorithms may fall into more than one category. At the end of this section, we present some suggestions for further research in the cognitive neuroscience of MTT.

#### Attention-based MTT methods

Humans are unable to process all incoming visual information simultaneously. To overcome this limitation, they use attention as a mechanism to select what to process. The overtly attended region is projected onto the fovea (a part of the retina with the highest spatial acuity and the highest density of photoreceptors), and the surrounding areas are projected onto parts of the retina that are far from the fovea, which have a lower density of photoreceptors. Many researchers have studied attention, and different models have been proposed to predict which part of an unseen image will be attended to by humans. Many attention models have been developed in the past, and reviewing them all goes beyond the scope of this work. Refer to [94–96] for comprehensive reviews on attention modeling.

A fundamental concept in modeling attention is a center-surround mechanism, suggesting that an item that is highly different from its surround is salient and attracts attention. This mechanism has been used in several algorithms. For example, authors in [23,97] utilized this mechanism to propose a discriminant tracker for a single object. They chose and used features with the highest ability to discriminate a target from its surrounding background from a feature pool. For this purpose, they applied features on both center and surround. Then, the Kullback–Leibler divergence between probability distributions of center and surround is calculated to measure the ability of features that discriminate these two. In [38], another strategy is proposed to benefit from both center and surround information to facilitate tracking. The author uses a swarm of tracking windows, each capable of tracking a part of a single moving target. Windows are assigned to corners of a target after applying a corner-detection algorithm in the initialization phase. Therefore, some windows contain some parts of the target, and others contain its surround. Windows are tracked separately, and the patch with the highest normalized cross-correlation (NCC) is considered to be the new position of the target. Motion direction, speed, and the size of the target were also inferred from corners. Target motion is calculated according to mean motion of windows. To handle changes in target size, the associated motion of windows in right versus left and top versus bottom is examined. Changes in swarm motion can be interpreted as changes in size. Outlier windows that have inconsistent motion regarding swarm motion are dropped, which reduces window population after several steps. In this case, swarm is reassigned to the targets. This study uses corner features of objects to follow their changes in size. However, in cognitive studies of MTT while subjects are instructed to track targets in a 3D environment, it is an open question how they treat changes in targets’ size due to perspective.

Attentional mechanisms are usually characterized as either bottom-up or top-down processes. Bottom-up attention is stimulus driven and unconscious, whereas top-down attention is goal driven and conscious. Some algorithms use these mechanisms to develop search capabilities. They usually use bottom-up attention to extract features and top-down processes in order to find the target’s new position (the most similar object according to extracted features) in the new frame. Features extracted in the bottom-up phase can be discriminant features [23,97]. In the top-down phase, some algorithms search the neighborhood of the current position of the target in the next frame to find the most similar part. These two steps are repeated one after another, which means that target features are updated in their new positions to easily track appearance changes in the target. In other words, in such discriminant trackers, there is less bias towards using target representative features. Instead, features with a high ability to discriminate between the target and background are used, which is useful for handling both the target`s variable appearance and variable background challenges. For tracking multiple targets, this strategy is extendable.

Another method considers spatiotemporal attention mechanisms using a framework based on dynamic CNNs for MTT [98]. This method solves two MTT challenges. Using a dynamic CNN helps handle computational complexity, and using spatiotemporal attention helps avoid missing objects in case of the occlusion of multiple targets. The CNN-based framework has some shared layers and devotes a separate branch to each tracker. The shared layers encode the features of the whole frame and do not change during the tracking process. The branches have the same structure, while each one is trained on a separate object as an SOT; a new branch is devoted to a newly defined target, and, when a target is removed, its corresponding branch is removed as well. Each branch consists of a visibility map that determines nonoccluded regions, an attention map to weigh the feature map, and a binary classifier to separate the object from its background. This appearance model is trained online using new and historical samples via the backpropagation algorithm. Temporal attention is also considered to weigh the previous correctly detected samples against the new ones according to the amount of occlusion. The more a sample is occluded, the lower its weight.

Authors in [99] focused on spatiotemporal continuity by checking for inconsistencies in spatial coherence according to psychological findings suggesting that spatial information plays a dominant role in preserving target identity. Their method considers potential associations of targets in the spatial domain and evaluates appearance, motion, and other features in the visual domain as inspired by the famous two-stream hypothesis about what (object) and where (location) attentional pathways [100].

Among different approaches reviewed in [101], attention provides a suitable method for recognizing targets by discriminating them from the background and from each other. This helps handle important challenges, such as variable target appearance and background variability. But, due to its adaptability to variation, it suffers from occlusion. When a target is gradually occluded by an occluder, the algorithm may tend to accept it as a change in target appearance and thus miss the target. A successful solution to avoid this involves using memory to retain appearance information of the targets and limiting the range of variations in target-appearance changes.

#### Memory-based MTT methods

Memory is the second cognitive mechanism with significant impact on MTT performance. To find the position and identity of targets at each moment, one needs to remember them. This helps to overcome occlusion challenges. In the case of partial occlusion, it helps to detect the parts that belong to the target and to exclude the occluder. In the case of complete occlusion, after reappearance of a target from a complete occlusion, we can remember which object was the target. Memory also helps retain different appearances of a target and overcome the variable target-appearance challenge. According to the Atkinson–Shiffrin memory model (ASMM) [102], there are three memory stages: sensory memory, short-term memory, and long-term memory. A study used this model to handle sudden changes, such as illumination changes and occlusion, in the proposed MTT algorithm [103]. The model is based on compressive tracking that uses sparse and reduced discriminant features to find the target in a new frame. The authors also applied a weighting method to emphasize the nearer candidates for targets (according to the euclidean distance between the target in the previous frame and the candidate in the new frame) and to lower the contribution of others. This approach leads to higher accuracy when the target is similar to the background.

In [104], authors used the aforementioned memory model to propose a single target MUlti-Store Tracker that can memorize target appearance. This method supports both translation and scale invariance. A forward-backward tracker [105] is applied to two consecutive frames to identify any failure in tracking, i.e., the tracker estimates the target position in the first image according to its determined position in the second image. The amount of displacement is expected to be small. Comparing the estimated position with the real position identifies the best key points to successfully track the target; key points are used to find the best match for the target, to determine its position in the new frame according to the euclidean distance, and to reject outliers. Occlusion handling is also possible. A small number of key points matching the background in the targets’ bounding boxes means that no occlusion is happening. As the number of key points matching the background approaches the number of target key points, the target is more occluded. The target is remembered and stored in short-term memory when it is not occluded.

Forgetting is also used for descriptors inspired by how humans lose information when there is no attempt to retain it. As a feature is recalled more, forgetting becomes harder. Another memory-based SOT is proposed with adaptability to variation in target appearance and environment [106]. This proposed memory model has three components: ultra-short-term memory, short-term memory, and long-term memory, each associated with the processes of encoding, forgetting, and remembering. Both of the above-mentioned algorithms can be extended to track multiple targets. Targets and background usually change over time in a natural environment. A challenge for computer-vision algorithms is successfully keeping track of targets in these situations. On the other hand, stimuli used in cognitive studies of MTT are too simple and lack any changes that may happen naturally. A productive area for future study is how humans deal with this challenge in MTT.

The memory module in an MTT algorithm is useful for handling challenges in target tracking, such as occlusion, variability in target appearance, and data association. In tracking more than one object, the role of memory becomes even more important. However, a memory-based algorithm needs to establish some critical parameters, such as what to remember and when to forget something. The importance and necessity of memory in algorithms motivated some studies (in particular, those using deep neural nets as their main tool) to propose LSTM (Long–Short-Term Memory) networks.

#### MTT methods based on ANNs

ANNs are commonly used in computer vision. They are inspired by the human nervous system; each neuron in an ANN receives inputs from one or more neurons, applies a simple function on the weighted sum of inputs, and emits its output to others. Different attempts have been made to improve neural nets according to biological neural-processing evidence. These efforts have led to convolutional neural nets, recurrent neural nets, and spiking neural nets as well as networks with various architectures (e.g., the Hopfield network).

In the context of tracking, neural nets have been widely adopted. In [107], a five-layer network inspired by the primary visual cortex is proposed. Three layers perform object recognition and present a biologically inspired appearance model. The remaining two layers strengthen the discriminative ability of the model in distinguishing the target from the background. This architecture also includes layers for whitening, coding (which uses discriminative dictionary learning methods), rectification, normalization, and sum pooling (making the model find global features). This network was proposed to model appearance when a particle-filter algorithm is used for tracking. The weights for particles are calculated according to the similarity of the learned target and candidates. The particle with the highest weight is considered to be the tracking result in the current frame. To handle target variation during the video, a set of target templates are used, and a weight is assigned to each one to define the current appearance of the target. Authors in [108] postulated that having a large amount of auxiliary data is enough to learn a CNN (in an online manner) that can track a target robustly without offline training. They proposed a lightweight, two-layer feedforward convolutional network tracker (CNT) accordingly and ignored pooling layers in the architecture of their net in order to keep high-resolution spatial features and their precise positions. A particle filter then estimates the exact position of the target in the new frame.

The methods mentioned above are all SOTs. Neural-net–based algorithms for MTT, however, are rare. For example, [109] benefits from the motion-history image, which is the difference between the current frame and historical images. However, their approach cannot detect stationary objects. Therefore, another feature is also used to strengthen object detection and recognition. A number of filters are learned using a convolutional restricted Boltzmann machine in an unsupervised manner. Using features and motion-history information, a feature map is produced for each frame. Using this method, all objects in the scene are tracked, and this information is used to detect and recognize objects in a scene.

Another study proposed an end-to-end recurrent neural network (RNN) approach with an LSTM for MTT that can handle various challenges, especially the unexpected entrance or exit of objects, which happens in a real scenario [110]. This is a challenge for algorithms that is ignored by almost all cognitive studies of MTT. The RNN is responsible for temporal prediction, and the LSTM is responsible for handling data association. The proposed method is entirely data driven and does not need any prior knowledge. Required data is generated by sampling from a generative model. A multidimensional state space is used concurrently to determine the states of all targets with different types of variables and desired numbers of outputs, each corresponding to a single target. In robotics, authors in [91] used an RNN (particularly, a deep LSTM network) with the help of heading estimation and spatial information to identify robots with the same appearance in robot collaboration tasks.

Recently, deep reinforcement learning (DRL) has also been shown to be an innovative approach for demonstrating object localization, active object tracking, and MOT [111,112]. For example, [113] used a DRL method to locate targets in the new frame considering the collaborative interaction of targets and environment based on detection results of targets in the previous frame. For this purpose, they combined a prediction network (responsible for predicting the location of targets) and a decision network (responsible for feature extraction and finding optimal results regarding interaction of targets and the environment). In this way, they initialize, delete, or update information related to all targets in each frame.

In general, ANNs have the potential to apply to cognitive mechanisms and are shown to be effective for tracking more than one object. However, problems remain (e.g., the requirement for a large amount of data, instability, etc.), which sometimes makes ANNs unsuitable for some real-world scenarios.

### Discussion

MTT algorithms are mainly used in real-world complicated applications, and they are mismatched to cognitive MTT tasks, which are designed as simply as possible. Studying human behavior while tracking multiple objects in real-world experiments helps to unearth strategies normally used by humans. It is common in algorithms to handle objects’ scale changes. However, to the best of our knowledge, scale changes are studied in general [114], not specifically in the context of cognitive research of MTT. Using memory is an approach for occlusion handling in computer-vision algorithms. This raises the question in the cognitive science of MTT studies about whether subjects with better working memory (or short-term memory) perform better at tracking multiple objects through occlusion. Answering this question will inform us about the mechanism behind occlusion handling in humans; whether it is memory or something else remains to be discovered. Another common challenge for MTT algorithms is continuous changes in the appearance of targets and background, which can make it difficult to track targets; this raises the question of how humans tackle this problem.

Occlusion of targets or target distractors is common in real-world applications. However, most MTT cognitive research has studied the effect of occlusion due to solid occluders such as walls or environmental factors. Studying occlusion due to other moving objects (targets or distractors) can be useful in this domain. In computer-vision algorithms, it is common to use the exact surround of the targets for discrimination. Do humans use the same strategy to track objects in real-world and natural scenarios when the background is not as simple as the artificial stimuli used in most MTT cognitive neuroscience studies? On the other hand, a challenge in computer-vision algorithms is how they handle unexpected entrance or exit of a target from the scene. To the best of our knowledge, this topic has not been studied thus far; however, answering this question is necessary for understanding the exact mechanism behind MTT used by human subjects. Finally, it is demanding to identify to what extent the two approaches of discriminant- and detection-based trackers contribute to daily MTT activities. Brain-imaging technologies can help us in this regard and show more detailed information about the neural mechanism underlying MTT in the brain. We can compare how brain parts responsible for object detection are involved in both MOT and MIT.

## General discussion

MTT is an important paradigm in neuroscience. It is also essential for a variety of applications in computer vision. A line of research in this area is concerned with learning from the brain to build and improve the accuracy of algorithms in computer vision. These studies have led to three types of brain-inspired methods: 1) attention based, 2) memory based, and 3) neural-net based. According to cognitive studies, memory and attention are cognitive processes that are clearly involved in MTT [22]. Tasks measuring visuospatial short-term and working memory as well as attention switching proved to be significant predictors of MTT. Thus, tracking is not automatic, and keeping track of targets demands attentional resources [115]. In fact, the inspiration behind these computer-vision models indirectly imitates brain function in general, although the implementation details are not exactly in line with how the brain tracks multiple targets. Using ANNs as an implementation tool can be beneficial since its building blocks (e.g., neurons) imitate the human nervous system and thus have the potential to encompass the aforementioned cognitive processes. With regard to studies related to MTT in neuroscience, there are some commonalities as well as incongruities between computer vision and neuroscience, which we discuss below.

### Neuroscience and computer-vision commonalities in MTT

Researchers in both neuroscience and computer vision believe that attention and memory are critical in MTT. In neuroscience, many studies have proven the importance of these factors [12,22,24,26,37,47,65,69]. In computer vision, different algorithms have been applied that benefit from the role of attention and memory in increasing the accuracy and performance of their methods [38,98,99,103,104]. Tracking algorithms in computer vision are commonly divided into two groups: (1) algorithms that use a tracking-by-discrimination approach and (2) algorithms that follow a tracking-by-detection approach. Some algorithms in the first group utilize brain-inspired methods that benefit from center-surround saliency to discriminate the target from background [23,97]. This is in line with the saliency hypothesis in tracking, which states that a salient item can be tracked with higher accuracy than a nonsalient one [116]. In tracking-by-detection methods, on the other hand, an object-detection module is usually included, which detects all related objects in the scene [117–119]. The tracking module considers different locations of detected objects in successive frames to determine the object’s trajectory. The advantage of this approach is handling unexpected entrances or exits of a target.

Although we include tracking by discrimination as a brain-inspired strategy, some evidence in neuroscience suggests that tracking by detection is also a strategy used by the brain. In fact, evidence shows that tracking and identity processing share the same attentional resources, especially in the case of face tracking [65]; some neuroimaging studies have reported activation of areas responsible for object recognition in the brain during MTT [24]. We therefore hypothesize that the brain uses both strategies. To have a realistic brain-inspired MTT algorithm in the area of computer vision, one could benefit from a combination of these approaches. It is also common to use a motion module to predict the trajectory of targets in MTT computer-vision algorithms. This is in line with studies in neuroscience that provide evidence for subjects using motion information and extrapolation to track objects [120,121]. Neuroscience and computer-vision studies both agree on the general framework of MTT, which emphasizes the importance of attention, memory, and motion prediction. But there are few or almost no practical algorithm in the computer-vision domain that uses all the aforementioned modules as its building blocks.

### Neuroscience and computer vision inconsistencies in MTT

Although the fields of neuroscience and computer-vision believe in the same framework for mechanisms underlying MTT, there are many differences in the approaches used in these two areas. For example, studies suggest that any visual separation between targets and distractors, which can be due to surface features or semantic information, can streamline perceptual grouping and improve the performance of subjects during tracking [25–28]; however, no computer-vision algorithm has utilized this finding as inspiration behind its methods. This could be incorporated by processing semantic and surface information of targets in an object recognition module in MTT algorithms, which may improve tracking performance. Computer-vision distractors are not explicitly defined; rather, algorithms mainly focus on discriminating targets with their surrounding background. One challenge, then, is how to determine the correct border to separate objects from the background. Algorithms are highly likely to introduce a window that is half target and half background, for example. However, humans are able to introduce objects effortlessly. In cognitive studies, people introduce distractors as an object when making mistakes.

While practical tracking algorithms include a motion-prediction module, the question of “whether humans use motion prediction during tracking or not” is still open in the field of neuroscience. Some studies investigated the behavior of subjects during the tracking of objects that undergo occlusion in the middle of their trajectory or disappear for a short period of time and reappear. They suggest that subjects use this motion-prediction information in object tracking after occlusion [69]. There is also evidence supporting the idea that no prediction occurs during tracking of more than two targets. For example, in [122], subjects are asked to track several circles out of eight circles filled with four colors (yellow, red, blue, and green). On each trial, there was a target and distractor of each color. At the end of the tracking phase, the cursor turns into one of the targets appearing at the screen’s center. Participants have to position the disc (using a mouse cursor) at the last location of the queried target. They reported that perception lagged rather than anticipated future positions and results were biased towards lagging positions. Although several factors might lead to a position lag, it might be a result of temporal integration of visual signals, serial attention, and encoding into short-term memory. This shows that a memory module might be more critical than a motion-prediction module for computer-vision algorithms. It is reported that tracking performance is always more accurate when objects appear at their disappearance loci than at their predicted positions [61]. Thus, understanding the exact mechanism behind the brain can inspire algorithms that currently utilize a motion-prediction module.

Although we included ANNs as a powerful tool for developing MTT algorithms, their functional details fundamentally differ from biology. Cortical neurons in the brain are organized in a goal-driven manner, each with particular functions, and they communicate to each other in different ways. The process proceeds dynamically and evolves over time. Further, unlike ANNs, no separate training phase is needed. Methods based on ANNs rarely match the true approach that the brain uses. Some existing methods assign a particular part of the network to a particular target to track, but no evidence shows that the brain behaves in the same way.

The main challenges that target-tracking algorithms face include variability in target appearance and background, partial or complete occlusion, and data association. It seems that the brain handles these challenges more successfully and rapidly. Although it is still not clear how the brain performs exactly, cognitive studies of MTT have been successful to date in discovering some aspects. Even this incomplete information can improve both the accuracy and runtime of MTT algorithms.

Another challenge in computer-vision studies that has not been addressed in the field of neuroscience is handling the birth and death of a target. Paradigms used by neuroscientists to study human MTT are more simplistic than those used to test computer-vision algorithms [5,123]. To study the fundamental aspects of MTT, neuroscientists present simple artificial scenarios to subjects. However, using stimuli with greater ecological validity is also important and may implicate additional brain areas involved in real-world MTT that are not active when viewing synthetic stimuli.

Human vision is binocular, and it has been reported that humans perform better when depth information is also available in MTT stimuli. However, there are still open questions about how and to what extent human binocular or even monocular depth vision influences the handling of challenges such as clutter, scale variance, and data association or occlusion. Most of the stimuli used in MTT cognitive experiments are 2D; this raises the issue of circumstances in which human tracking of performance using binocular vision is better than monocular. Most MTT algorithms are based on monocular vision mainly due to economic or technological constraints or even due to the lack of knowledge of developers concerning its role in tracking performance. Indeed, this issue has rarely been considered by both communities.

Another problem faced by computer-vision–tracking algorithms is multiple-class MTT or tracking different objects from various categories, which is done easily by humans. It is difficult for a computer-vision algorithm, especially one with a tracking-by-detection strategy, to manage this since these types of methods need one or more (pretrained) algorithms with the ability to detect different types of targets. Thus, gaining inspiration from the brain about how to track objects from multiple classes could be highly rewarding in improving artificial tracking algorithms.

So far, we encourage computer-vision researchers to motivate mechanisms underlying MTT algorithms based on existing knowledge as to how the brain processes similar tasks; the hope being that this insight might improve the tracking performance of these algorithms. In many cases, the advantages of the strategies used by the human brain to efficiently track multiple targets can be used as inspiration behind computer-vision algorithms. However, several processing limitations humans face during MTT are not inherently limiting to computer-vision models. Computer vision itself has some advantages over biological vision. For example, in computer vision, we might utilize multiple cameras to monitor a scene from different angles of view, and some algorithms benefit from this to handle occlusion. Thus, all parts of an image can be processed in high resolution with great detail. A human is unable to do so because the brain is inherently limited in processing the sensory information it receives and the eye is physically limited by being able to capture only a part of the visual field with high resolution at any given time. Thus, from a pure perceptual standpoint, changing the focus of attention to capture the whole visual scene in the way a human does is irrelevant to computer-vision approaches. However, investigating how humans organize and retain the information they perceive from viewing different parts of a scene can be helpful towards applying the same strategy to multicamera problems in computer vision.

Another limitation faced by the human brain in MTT is the maximum number of targets being tracked. This number is limited in humans because of capacity limitations in working memory to encode the location and appearance of targets and limitations in attentional capacity to attend to more than a certain number of stimuli [124]. However, for computer-vision algorithms, such limitations are not faced by the system since we can enhance the storage space or processing capacity using more powerful hardware and software.

## Conclusion

In this paper, we reviewed MTT studies both in the areas of computer vision and neuroscience and discussed their commonalities and inconsistencies. Despite significant efforts, several unresolved points still exist pertaining to MTT that are worth noting. For example:

- From cognitive studies, we know that the human brain utilizes all available information to discriminate among objects. Using depth information, binocular vision and information about the intrinsic motion of nonrigid targets is expected to be beneficial in improving the performance of MTT algorithms.
- As a successful strategy for MTT, human subjects consider targets as vertices of a polygon and follow its center. Considering spatial configuration of targets (or objects in general) while estimating target loci empowers the algorithms and improve their accuracy in handling partial or complete occlusion in some applications, e.g., tracking vehicles in traffic-monitoring systems.
- Motivated by the parallel strategy used by the brain to track similar objects and considering the power of computer vision to assign enough computational resources, parallel programming can increase performance of MTT algorithms. In such algorithms, all targets can be tracked in parallel to improve runtime.
- A controversy remains in cognitive studies about whether the human brain uses objects’ motion information (or extrapolates it) to handle occlusion. Understanding the exact mechanism or the situations in which each is used would help us improve related machine-vision algorithms.
- The question of how the human brain handles the entrance of a new target or exit of an existing one in MTT tasks has not yet been studied. Almost all existing studies considered a fixed number of targets and distractors in their design.
- It is more valuable and inspiring to study human behavior and brain activities while performing real-world experiments than common synthetic scenarios.

Overall, computer-vision algorithms struggle with important challenges that can be studied in neuroscience to identify strategies the brain uses to resolve similar problems. Similarly, important findings in neuroscience related to MTT can improve the efficiency and accuracy of computer-vision algorithms. We suggest strengthening the connection between these two fields to develop more powerful MTT computer-vision algorithms and to better understand the mechanisms supporting MTT in the human brain.
